# Immune Sensing of *Candida albicans*

**DOI:** 10.3390/jof7020119

**Published:** 2021-02-06

**Authors:** Ebrima Bojang, Harlene Ghuman, Pizga Kumwenda, Rebecca A. Hall

**Affiliations:** 1 Institute of Microbiology and Infection, School of Biosciences, University of Birmingham, Birmingham B15 2TT, UK; EXB862@student.bham.ac.uk (E.B.); HXG051@student.bham.ac.uk (H.G.); PXK603@student.bham.ac.uk (P.K.); 2 Kent Fungal Group, Division of Natural Sciences, School of Biosciences, University of Kent, Canterbury CT2 7NJ, UK

**Keywords:** *Candida*, cell wall, neutrophil, macrophage, candidiasis

## Abstract

*Candida albicans* infections range from superficial to systemic and are one of the leading causes of fungus-associated nosocomial infections. The innate immune responses during these various infection types differ, suggesting that the host environment plays a key role in modulating the host–pathogen interaction. In addition, *C. albicans* is able to remodel its cell wall in response to environmental conditions to evade host clearance mechanisms and establish infection in niches, such as the oral and vaginal mucosa. Phagocytes play a key role in clearing *C. albicans*, which is primarily mediated by Pathogen Associated Molecular Pattern (PAMP)–Pattern Recognition Receptor (PRR) interactions. PRRs such as Dectin-1, DC-SIGN, and TLR2 and TLR4 interact with PAMPs such as β-glucans, *N*-mannan and *O*-mannan, respectively, to trigger the activation of innate immune cells. Innate immune cells exhibit distinct yet overlapping repertoires of PAMPs, resulting in the preferential recognition of particular *Candida* morphotypes by them. The role of phagocytes in the context of individual infection types also differs, with neutrophils playing a prominent role in kidney infections, and dendritic cells playing a prominent role in skin infections. In this review, we provide an overview of the key receptors involved in the detection of *C. albicans* and discuss the differential innate immune responses to *C. albicans* seen in different infection types such as vulvovaginal candidiasis (VVC) and oral candidiasis.

## 1. Introduction

*Candida albicans* is both a commensal and opportunistic fungal pathogen of humans, the delicate balance of which is maintained by the actions of the innate immune system and resident microbiota. *C. albicans* is able to colonise most areas of the body, including the nails, skin, mucosal membranes, and internal organs. The colonisation of the skin and gastrointestinal tract is thought to occur at birth when the infant passes through the birth canal, where *C. albicans* is a natural resident [1]. During periods of immune suppression, such as in those with HIV, neutropenia, or dysbiosis of the microbiota, *C. albicans* can overcome the innate immune system to establish various infections, such as superficial mucosal infections (oral and genital thrush), subcutaneous infections and systemic disease (candidemia). Although mucosal infections are not life-threatening, they pose high morbidity rates and are expensive to treat, while candidemia is associated with a poor prognosis and high mortality (40–50%) [2]. *C. albicans* is armed with a plethora of virulence factors including adhesins (i.e., Hwp1, Als3, and Als5), secreted hydrolytic enzymes (i.e., SAPs and phospholipases) and the recently identified cytolytic toxin candidalysin [3,4]. *C. albicans* can also form biofilms in nail infections; on teeth exacerbating, tooth decay; on mucosal surfaces; and on the surfaces of medical devices, including catheters, long lines and voice prostheses [5,6]. This capacity to form biofilms has made *C. albicans* one of the leading causes of nosocomial infections, which are difficult to treat due to increasing antifungal resistance.

Despite the causal agent being the same in each infection, the innate immune responses elicited during these infections are very different, suggesting that the host environment plays a key role in modulating the host–pathogen interaction. The majority of innate immune responses are mediated by the interaction between Pattern Recognition Receptors (PRRs) expressed on the surface of innate immune cells, and Pathogen Associated Molecular Patterns (PAMPs) on the fungal surface. The *Candida* cell wall consists of an inner skeletal layer of β-glucan and chitin, and an outer layer of glycosylated proteins [7,8]. These proteins play essential roles in adhesion to host cells, while carbohydrate PAMPs are recognised by PRRs on the surface of innate immune cells. The main families of receptors involved in the recognition of *C. albicans* are the C-type lectin receptors (CLRs), RIG I-like receptors (RLRs), NOD-like receptors (NLRs) and Toll-like receptors (TLRs), which recognise distinct PAMPs (Table 1). Each innate immune cell has a distinct, but overlapping, repertoire of receptors, giving rise to its preferential recognition of certain morphotypes. The response elicited against a microorganism is governed by the mosaic of receptor complexes engaged. In this review, we provide an overview of the key receptors involved in the detection of *C. albicans*, summarise the immune sensing of *C. albicans* occurring in different infection types, and discuss the potential role the host environment plays in modulating these responses.

## 2. Recognition by Phagocytes

The successful clearance of *C. albicans* from the host tissue relies heavily upon the phagocytosis of the fungal pathogen by innate immune cells (i.e., macrophages, neutrophils and dendritic cells). Phagocytosis is a multi-step process initiated by pathogen recognition. This leads to the engulfment of the pathogen, phagosome maturation to eliminate the pathogen and then the resolution of the phagolysosome, and this has been extensively reviewed by Levin et al. [26]. Innate immune cells interact differently with the yeast and hyphal morphologies of *Candida* [27]. This is partly due to the differential exposure of β-glucan, with β-glucan exposure at yeast bud scars provoking Dectin-1-dependent proinflammatory innate immune responses [11]. Hyphae generally have less β-glucan exposed on their cell surface and, therefore, are less effectively recognized by Dectin-1 [11]. However, activated neutrophils can expose β-glucan in hyphal cells, leading to Dectin-1-dependent recognition and proinflammatory cytokine secretion [28]. Furthermore, the hyphal orientation during contact with phagocytic cells also impacts phagocytosis, with macrophages encountering hypha tips phagocytosing the *Candida* quicker [29]. Therefore, there are multiple factors that contribute to how macrophages interact with *Candida*. Dendritic cells (DCs) also discriminate between the different morphologies, and this differentially stimulates adaptive immunity, with yeast cells provoking a Th1 response, while hyphae initiate a Th2 response [30].

**Figure 1:**
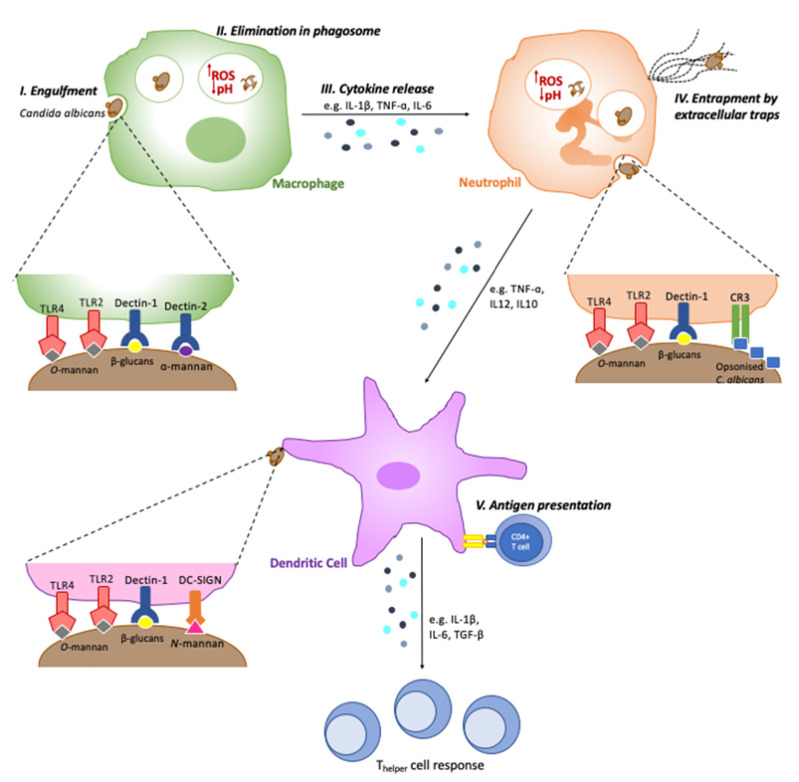
Key response pathways activated by innate immune cells against *Candida albicans*. Following successful recognition of *Candida* PAMPs (such as *O*-mannan, α-mannan, *N*-mannan and β-glucans) by immune cell PRRs (such as TLR2, TLR4, Dectin-1 and DC-SIGN), immune cells use a number of response mechanisms to deal with *Candida* infections. These include (I) engulfment and (II) elimination of the pathogen within the phagosome, where it encounters acidic pH and increased reactive oxygen species (ROS) production; (III) cytokine release, aiding immune cell recruitment, and exerting direct antimicrobial effects; (IV) entrapment and elimination of pathogens by releasing extracellular traps comprised of DNA with antimicrobial agents; (V) antigen presentation, activating the adaptive immune system by presenting antigenic peptides to T-cells on their major histocompatibility complex (MHC) molecules.

Neutrophils are the major immune cells in the control of candidiasis, and thus, neutropenic individuals and mice have an increased susceptibility to candidiasis [31,32,33,34]. The C-type lectin receptor Dectin-1 is the most important neutrophil PRR in *C. albicans* recognition. The engagement of Dectin-1 leads to the activation of integrin Mac-1 (CR3), and both receptors are essential for fungal engulfment and elimination [12]. Macrophages also play a key role in phagocytosis and *Candida* clearance in various niches, while dendritic cells specialise in processing and presenting antigenic material from *Candida* to lymphocytes, acting as a bridge between the innate and acquired immune systems [35]. Like neutrophils, these innate immune cells recognise *C. albicans* by its cell wall PAMPs; β-1,3-glucan is recognised by the C-type lectin receptor Dectin-1 and complement receptor 3 (CR3); *N*-mannan, by Dectin-2, DC-SIGN and MINCLE; and O-mannan, by TLR4 (Table 1 and Figure 1) [14,36,37,38,39].

The recognition of these PAMPs initiates phagocytosis, which involves two main stages: (i) phagosome formation and (ii) maturation. Phagosomes are formed when phagocytic receptors such as CR3, Fcγ receptors (FcγRs) and Dectin-1 bind to phagocytic targets and result in receptor clustering. Subsequent to receptor clustering, the phosphorylation of their immunoreceptor tyrosine-based activation motifs (ITAMs) and the activation of Rho-family GTPases occur. Rho-GTPases activate nucleation-promoting factors (NPFs), which cause actin-driven protrusions from the plasma membrane to advance around the target [40,41]. The activation of myosin by cytosolic Ca^2+^ has been suggested to be necessary in phagocytic cup formation and sealing, to form the phagosome [26,42].

Phagosome maturation is a gradual process whereby the phagosome is converted into a hostile and degradative environment to achieve pathogen elimination. The process differs between neutrophils and macrophages, which has been reviewed in detail [26,43]. Briefly, in neutrophils, phagosome maturation occurs rapidly and involves fusion with lysosomes and neutrophil granules containing antimicrobial peptides and proteolytic enzymes [43,44,45]. In macrophages, endosomal fusion begins early during phagosome maturation and is driven by the recruitment of active Rab5 and then Rab7 as they transition into late phagosomes. Eventually, lysosomes fuse with late phagosomes to form a phagolysosome, which is armed with lytic enzymes and is highly acidic (i.e., in M2 macrophages, which are involved in tissue repair and the resolution of inflammation) and oxidative, to provide an extremely degradative environment for pathogens [26,46,47,48]. Notably, neutrophils and M1 macrophages (proinflammatory macrophages)—the cells mainly responsible for pathogen elimination—maintain a slightly alkaline compartment during this transition. These phenomena are attributed to the increased proton consumption during superoxide dismutation, reduced granule fusion observed during reactive oxygen species (ROS) production, and increased leakiness for H^+^ caused by the oxidative effect of ROS [49,50]. The sustained alkalinization of the phagosomal compartment is also true for dendritic cells. Dendritic cells prioritise antigen presentation and, therefore, must regulate their phagosomal contents for partial degradation [51]. To achieve this, ROS production is maintained at low levels for sustained periods by the recruitment of NADPH oxidase, NOX2, to phagosomes shortly after phagocytosis [51].

ROS generation is initiated by NADPH activation in neutrophils and potentiates *Candida* killing within phagosomes [39,52,53]. In neutrophils, NADPH is produced by two separate, glucose-dependent pathways: glycolysis and the pentose phosphate pathway [54]. Kidney patients with reduced expression of a glucose transporter, GLUT1, and patients with chronic granulomatous disease have increased susceptibility to candidiasis, exemplifying the importance of NADPH oxidase [55]. In macrophages, the engagement of Dectin-1 causes the phosphorylation of the ITAM-like motif in its cytoplasmic tail. This leads to the subsequent recruitment of the tyrosine kinase, Syk, and the activation of NF- ϰB, which is essential for proinflammatory cytokine production, phagosome acidification and ROS generation [56,57,58,59]. Together, these stresses eliminate and dispose of *C. albicans*. However, achieving this end goal is made difficult by the morphological change that occurs in *C. albicans* as they transition from yeast to hyphae. This imposes mechanical stress on the phagosome and may lead to phagosomal rupture, although other factors such as candidalysin release have also been implicated in phagosomal rupture and escape [60]. To circumvent this problem, macrophages increase lysosome production upon the recognition and engulfment of *Candida*. These lysosomes are increasingly fused to the phagolysosomes to increase their surface area as the hyphae cause the distention of the phagolysosomal membrane, causing transient releases of Ca^2+^ into the cytosol. This ensures *Candida* remains confined within intact phagolysosomes long enough to effect killing [60].

The recognition of *Candida* by macrophages, neutrophils and dendritic cells, alike, results in the release of inflammatory and chemotactic cytokines, aiding further immune cell recruitment and infection resolution (Figure 1). *Candida* exposure results in the macrophage release of proinflammatory cytokines such as TNF-α, IL-1β and IL-6, enhancing *Candida* clearance and neutrophil recruitment [61,62,63,64]. Neutrophils play a key role in *Candida* clearance, by releasing proinflammatory cytokines such as TNF-α and IL-12, through the formation of neutrophil extracellular traps (NETs) and enhance dendritic cell recruitment and activation [65]. The release of IL-6 and IL-23 by dendritic cells augments the T_helper_ cell response to *Candida* infection, including the key T_h_17 response [66] (Figure 1). 

## 3. Niche-Specific Immune Sensing of *C. albicans*

### 3.1. Skin and Nail Infections

Warm and moist environments promote the expansion of *C. albicans* on the skin, leading to superficial skin infections, such as nappy rash, athlete’s foot and nail infections. *C. albicans* uses hydrolytic enzymes, such as secreted aspartic proteinases, shown to be important in tissue invasion and believed to be involved in establishing nail and other superficial infections [67,68,69]. Such infections are potentiated by increased phospholipase activity, which also correlates with biofilm formation, particularly in nail infections [67,69]. Although little is known about immune sensing in the skin and nails, the presence of DCs in the skin suggests their involvement in *C. albicans* sensing and the immune response in this context. Skin-derived DCs from foetal mouse skin phagocytose both yeast and hyphal forms of *Candida* [30]. The phagocytosis of the different morphotypes induces differential cytokine profiles, influencing the T-helper activation pathways. Yeast cells induce the production of IL-12 and activation of the protective Th1 pathway, while hyphae induce IL-4 production and Th2 activation [30,35]. Furthermore, the activation of the Th17 pathway in the skin requires the secretion of IL-1β and IL-6 [70]. This illustrates the discriminatory ability of DCs in sensing and responding to the different morphotypes of *Candida* (Table 2).

### 3.2. Oral Candidiasis

*C. albicans* is a commensal of the oral mucosa, but during periods of immune suppression, the balance between commensalism and pathogenesis is lost, leading to *Candida* overgrowth and infection. These infections are commonly associated with biofilm formation, especially in patients with dentures, voice protheses and xerostomia (reduced or absent saliva flow). Biofilm formation makes these infections difficult to treat because they are generally polymicrobial in nature, and the production of extracellular matrix increases the resistance to antimicrobial therapy [5,6,84]. Oral candidiasis is prevalent in immunosuppressed individuals such as HIV patients, mouth and throat cancer patients, and patients taking oral steroids making them highly susceptible to infection. Although the surveillance for oral candidiasis is limited, it is estimated that 20–50% of HIV patients develop oral candidiasis [85,86]. The prevalence of oral candidiasis in HIV/AIDS patients suggests that CD4^+^ cells play an essential role in combating oral colonisation, which is in contrast to the case for other *Candida* infections. 

**Figure 2:**
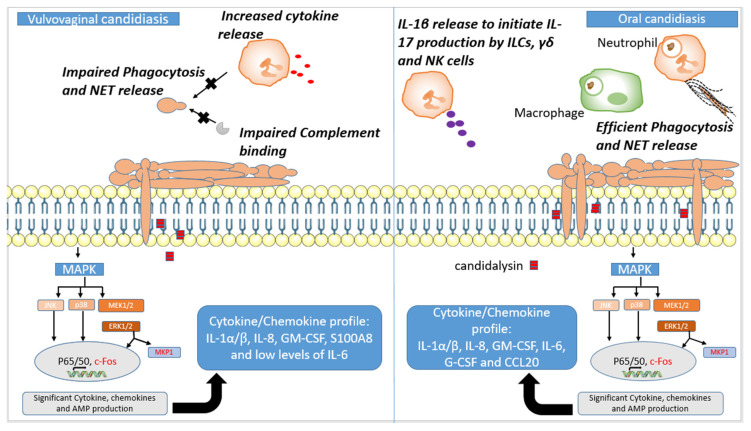
Host mucosal responses to *C. albicans*. Both vaginal and oral epithelial cells respond to *C. albicans* in a biphasic way. During colonisation, *C. albicans* only induces a mild response through MAPK signalling, and this does not result in cytokine release. However, a high burden of *C. albicans* coupled with hyphal growth and candidalysin release activates the second stage of the biphasic MAPK pathway, which involves activation of transcription factor c-Fos and MAPK phosphatase (MKP1), both depicted in red in this figure. MEK1/2 activates ERK1/2, which is the predominant regulator of MKP1. The activation of c-Fos, however, is predominantly regulated by p38. The ensuing cytokine profiles are distinct between the different sites as indicated in the boxes. This leads to immune cell recruitment to the mucosa. While this leads to the effective phagocytosis of *Candida* yeast and NET release to contain and kill hyphae in oral candidiasis, neutrophil recruitment causes the aggravation of disease in vulvovaginal candidiasis (VVC). In VVC, neutrophil phagocytosis is impaired, and this is partly due to reduced complement binding to *Candida*. This culminates in increased cytokine release, causing heightened nonprotective inflammation. In contrast to in VVC, innate lymphoid cells, γδ cells and natural killer cells play a vital role in *Candida* control by producing IL-17, known to potentiate the proinflammatory response of epithelial cells.

The immune sensing of *Candida* at the oral mucosa is initiated by the oral epithelial cells, by a biphasic MAPK response, which has been extensively reviewed elsewhere [87,88,89,90]. Briefly, low levels of *Candida* colonisation on the oral mucosa activate NF-ϰB signalling, Pi3K signalling and the three major MAPK pathways (p38, JNK and ERK1/2), through the recognition of components of the fungal cell wall (i.e., β-glucan and mannans) by traditional PRRs (i.e., TLR2, TLR4, EphA2 and Dectin-1) [91,92,93] as outlined in Table 1. However, this low-level activation does not result in a significant proinflammatory innate immune response, indicating that the recognition of cell wall PAMPs is not sufficient to fully activate oral epithelial cells. Instead, the induction of mucosal immunity requires the overgrowth of *Candida* on the oral mucosa coupled with hyphal formation. This increase in fungal burden on the oral mucosa activates the second MAPK response involving MEK1/2-ERK1/2, leading to the activation of c-Fos and MPK1—the “danger response” (Figure 2, Table 2). This strong activation of MAPK signalling results in the production of antimicrobial peptides, the secretion of proinflammatory cytokines, and subsequent phagocyte recruitment [91]. The activation of this second MAPK response is dependent on the secretion of the peptide toxin candidalysin [3,94], suggesting that epithelial cells primarily respond to damage rather than traditional PAMP–PRR interactions.

The recruited macrophages and neutrophils function to clear *C. albicans* through PAMP–PRR-directed phagocytosis, as detailed above, with NETs killing large hyphal cells that cannot be phagocytosed [95]. The subsequent production of TNF-α by phagocytes results in increased TLR4 expression on the epithelial cell surface, which confers mucosal protection [71]. In addition to the recruitment of phagocytes, the candidalysin-dependent activation of epithelial cells also recruits lymphoid cell type 3 (ILC3), γδ T-cells and natural Th17 cells, which are activated by IL-1β secreted by neutrophils and epithelial cells [74,75,76,96,97]. The activation of these cells results in the production of IL-17A and IL-17F, which potentiates the epithelial response by further inducing the secretion of proinflammatory cytokines, histatins and β-defensins from the epithelial cells and salivary glands [72,77,78,98,99] (Table 2). As a result, individuals with defective IL-17 signalling are highly susceptible to oral candidiasis, highlighting the importance of IL-17 in oral mucosal immunity to *Candida* [100,101].

### 3.3. Vaginal Candidiasis 

Vaginal candidiasis (VVC) is one of the most common *Candida* infections, infecting 75% of the female population, with up to 10–15% developing recurrent infections (RVVC), defined as three or more episodes in a twelve-month period [102,103]. Unlike for oral candidiasis, immune suppression is not a prerequisite for infection, and HIV-positive women are not more prone to the development of VVC. Instead, the predisposing factors include vaginal dysbiosis after antimicrobial therapy, elevated oestrogen levels (as a result of pregnancy, oral contraceptive use, or hormone-replacement therapy) and uncontrolled diabetes, and some single-nucleotide polymorphisms are enriched in women with RVVC [104,105,106,107,108,109]. Although it is a mucosal infection, the innate immune response in VVC is different to that in oral candidiasis, with IL-17 playing a minimal role [110,111,112]. 

Symptomatic vaginal infections are characterised by a nonprotective proinflammatory innate immune response, mediated by the influx of neutrophils, which has been reviewed in detail [113,114,115]. To summarise, the interaction of *C. albicans* with vaginal epithelial cells results in the production of IL-1β and S100A8 alarmin, which are potent neutrophil chemoattractants and function to recruit neutrophils to the vaginal epithelium [79,80,116] (Figure 2, Table 2). Neutrophil recruitment and, therefore, symptomatic disease are dependent on the production of candidalysin, with strains defective in candidalysin maintaining high levels of asymptomatic colonisation [94,117]. However, unlike in oral candidiasis, where neutrophils are efficient at phagocytosing yeast and trapping hyphae by NET release, these recruited neutrophils are unable to phagocytose and clear the fungal pathogen, leading to a heightened nonprotective proinflammatory response [79,118]. The neutrophil dysfunction in VVC and RVVC has been linked to the inability of complement receptor 3 (CR3) to interact with the fungal cell wall protein, Pra1 [119]. Under normal circumstances, the interaction of CR3 with Pra1 results in the formation of NETs and efficient killing of fungal hyphae [73]. However, this interaction is inhibited in vaginal fluid due to heparan sulphate, which competitively binds with CR3, preventing its interaction with Pra1, and promotes *Candida* survival and symptomatic infection (Figure 2) [115,120]. Heparan sulphate is induced by oestrogen, suggesting that women with high circulatory levels of oestrogen are more prone to symptomatic infection due to higher vaginal mucosal levels of heparin sulphate [121]. 

In addition to the role of host factors, *C. albicans* has been shown to remodel its cell wall in response to specific environmental cues encountered at the vaginal mucosa [122]. The growth of *C. albicans* in lactate—the predominant carbon source at the vaginal mucosa due to the presence of lactobacilli—promotes the concealment of β-glucans, reducing innate immune recognition and, therefore, possibly contributing to the reduced neutrophil phagocytosis rates [123,124]. On the other hand, acidic environments promote β-glucan exposure, leading to the hyperactivation of innate immune responses and significant neutrophil influx [125]. These acid-adapted fungal cells are more resistant to neutrophil killing and could also potentiate proinflammatory innate immune responses during VVC [126]. Analysis of the fungal cell wall from cells isolated from VVC patients identified β-glucan exposure on hyphae [127]. Neutrophils have been shown to expose β-glucan during NETosis, which is proposed to be the cause of glucan exposure on hyphae isolated from VVC patients, but given that neutrophils are defective in fungal killing at the vaginal mucosa, this remains to be confirmed [28,127].

### 3.4. Candidemia

In systemic infection, *Candida* enters the bloodstream and disseminates throughout the body. The presence of *Candida* in the blood is known as candidemia, the most common form of invasive candidiasis [128]. Candidemia can result in sepsis, infective endocarditis and vascular thrombosis, with often fatal outcomes [82]. In healthy individuals, *C. albicans* is rapidly removed from the bloodstream by the actions of neutrophils. Neutrophils are the most abundant leukocyte in the bloodstream, and whilst they are not essential in protection against other fungal pathogens such as *Cryptococcus*, neutropenic patients are more susceptible to systemic candidiasis. The chemokine receptor CXCR1 is essential in neutrophil granulogenesis, degranulation and non-oxidative killing of *C. albicans*, alongside the endoplasmic reticulum transmembrane protein Jagunal homolog 1 (JAGN1) [81]. The most infected organ in systemic candidiasis is the kidney, and neutrophils play a crucial role in host immunity to *Candida* [129]. In mice lacking the chemokine receptor CCR1, neutrophil accumulation in the infected kidney is impaired and delayed [130]. Interestingly, *ccr1^−/−^* mice display higher survival rates and lower kidney tissue damage, and *ccr1^+/+^* mice appear to succumb to uncontrolled tissue damage due to excessive neutrophil recruitment and activity, suggesting that the neutrophil CCR1 receptor mediates neutrophil tissue injury in invasive candidiasis [130]. Conversely, the absence of the chemokine receptor CXCR1 is associated with increased mortality and higher kidney fungal burden [131]. Neutrophil recruitment was not affected in *cxcr1^−/−^* mice, but the neutrophils displayed impaired degranulation and capacity to kill *Candida* [131].

The role of platelets in the innate immune response to *Candida* is often overlooked. Although traditionally described as playing a key role in maintaining haemostasis, thrombosis and inflammation, platelets have more recently been recognised as key players of the innate immune response to infection [132]. Whilst nonphagocytic, platelets have been shown to interact with and be activated by bacteria, viruses and fungi, leading to the release of various platelet antimicrobial peptides [133,134,135,136,137]. Thrombocytopenia is a known risk factor for invasive fungal infections, including candidiasis [136]. The *Candida*–platelet interaction is poorly understood and controversial, with the role of thrombosis in candidiasis yet to be determined. Several studies have shown that *Candida* species can bind and induce the activation of platelets *in vitro*, although the degree of this activation is strain and species specific [82,136]. Of those *Candida* species that can induce platelet aggregation, only small subpopulations of platelets have been shown to directly interact with *Candida* [136]. Additional studies have suggested that *C. albicans* might inhibit platelet aggregation through the production of gliotoxin, a fungal secondary metabolite, similar to *Aspergillus* [138]. However, the production of gliotoxin by *Candida* species has been debated [139]. The associated risk of candidiasis with thrombocytopenia highlights the importance of platelets as immune regulators in bloodstream *Candida* infection. However, current studies suggest that the *Candida*–platelet interaction is complex, and likely dependent on the platelet donor and fungal strain under investigation. Understanding the underlying mechanisms of the *Candida*–platelet interaction and the subsequent clinical outcomes surrounding the interaction could prove fruitful for developing novel antifungal therapies based on anticoagulants. 

In order to reach internal organs, *Candida* must cross the endothelium lining the blood vessels. To achieve this, *Candida* adheres to and transmigrates across endothelial cells. *C. albicans* express an array of adhesins that facilitate adherence to endothelial cells, such as α_M_β_2_-like adhesin, which interacts with endothelial ICAM-1 and -2; α_v_β_3_-like adhesin, which interacts with PECAM-1; and agglutinin-like protein 3, which interacts with *N*-cadherin [140]. The transmigration of *C. albicans* across endothelial cells is required for *Candida* to exit the bloodstream and enter the tissue. There are various models for how *Candida* transmigrates across endothelial cells. One model is that transmigration is facilitated by the endothelial endocytosis of *Candida*, mediated by the endothelial cell receptor *N*-cadherin [141]. Phan et al. showed that *C. albicans* hyphae bind to *N*-cadherin with greater affinity than yeast, and human CHO cells expressing *N*-cadherin endocytose significantly more *C. albicans* hyphae than CHO cells without cadherin expression [141]. *C. albicans* strains with homozygous null mutations in genes integral to filamentation regulation, such as *EFG1*, *TUP1* and *CLA4*, display impaired capacity to invade and damage endothelial cells [142]. Furthermore, the *∆tup1* mutant produced pseudohyphae on endothelial cells and displayed lower endothelial cell invasion and injury compared to the *∆cla4* mutants, which were aberrantly shaped but produced hyphae, collectively suggesting that the formation of true hyphae is required for endothelial invasion and damage [142]. Conversely, Shintaku et al. [143] showed that like that of *C. albicans*, the internalisation of *Candida parapsilosis* yeast cells is facilitated by N-WASP activation. However, *N*-cadherin does not appear to play a role in *C. parapsilosis* internalisation [143]. Whilst most *Candida*–endothelial cell interactions have been studied *in vitro*, there are also a few *in vivo* studies investigating the mechanism(s) underlying *Candida* adhesion to, and transmigration across, the endothelial lining of blood vessels. However, our current understanding of *Candida*–endothelial cell interactions must be validated *in vivo* and would benefit from the inclusion of other physical parameters including blood flow, which likely has an impact on the *Candida*–endothelial interaction.

## 4. Summary

Candidiasis encompasses a range of infections, such as low-mortality-associated infections such as oral thrush, and fatal infections such as candidemia. The ability of *Candida* to establish disease in host niche environments and our understanding of the mechanisms underpinning this are paramount in our bid to tackle candidiasis. The ability of the host immune cells—namely, but not solely, phagocytic cells—to identify and destroy *Candida* is key in our understanding of how the pathogenic fungus is able to evade immune clearance. Research on the interplay between *Candida* and phagocytic cells, such as macrophages and neutrophils, has alluded to the various PAMPs and PRRs involved in *Candida* detection. CLRs, such as Dectin-1 and DC-SIGN; TLRs, such as TLR2 and TLR4; and other receptors such as Galectin-3 and CR3 have all been shown to play a role in *Candida* infection. Likewise, corresponding PAMPs implicated in *Candida*–host interaction have also been identified, such as β-glucan and *O*-mannan. Whilst much has been done to identify which PAMPs and PRRs are involved in host–*Candida* interplay, there is much research to be conducted on the roles of these, and the subsequent activation pathways implicated in *Candida* engulfment and destruction, under the niche host conditions where *Candida* establishes itself. For example, under normal conditions, neutrophils are able to recognise, engulf and destroy *Candida* via the receptors CR3 and FcγR, and NADPH oxidase activity [144]. In VVC, however, there is significant neutrophil recruitment, but these neutrophils are unable to control the infection, and several environmental factors, including the heparan-sulphate-dependent inhibition of CR3 recognition and reduced Dectin-1 recognition due to lactate inducing β-glucan masking, have been suggested to account for this nonprotective innate immune response. Therefore, *Candida* appears to have a remarkable capacity to adapt to the host environment and subsequently evade clearance by the immune system. The key to developing therapeutics against candidiasis lies in our understanding of how *Candida* does this, and how it affects its interaction with immune cells and subsequent immune cell activity.

## Figures and Tables

**Table 1:** Pattern Recognition Receptors (PRRs) involved in the recognition of *C. albicans.*

Family	PRR	PAMP	Expression	References
*C*-type lectin receptors	Dectin-1	β-1,3-glucan	Dendritic cell, macrophage, neutrophil	[9,10,11,12]
	Dectin-2	α-mannan	Dendritic cell, macrophage	[13,14]
	Dectin-3	α-mannan	Macrophage	[15]
	DC-SIGN	*N*-mannan	Dendritic cell	[16,17]
	Mincle	α-mannosyl residues	Macrophage	[18]
	Mannose receptor (MR)	*N*-mannan	Dendritic cell, macrophage	[17,19,20]
Toll-like receptors	TLR2	*O*-mannan	Dendritic cell, macrophage, neutrophil	[21,22]
	TLR4	*O*-mannan	Dendritic cell, macrophage, neutrophil	[19,21]
	TLR6	Phospholipomannans	Macrophage	[23,24]
Complement receptors	CR3	β-glucans	Dendritic cell, neutrophil	[25]
Galectins	Galectin-3	β-mannosides	Macrophage	[10]

**Table 2:** Summary of immune sensing and responses at different infection sites.

Infection Type	Immune Sensing of *Candida albicans*	Inflammatory Response	References
Nail and Skin Infection	Dendritic cells	IL-12, IL-4, IL-1β, IL-6	[30,35,70]
Oral Candidiasis	Oral epithelial cells	IL-1α/β, IL-8, G(M)-CSF, CCL20, CXCL2, β-defensins, S100A8/9	[71,72]
	Neutrophils	IL-1β, NET formation	[73]
	ILC3	IL-17A/F	[74]
	γδ T-cells	IL-17A/F	[75]
	Natural Th17 cells	IL-17A/F	[76,77,78]
Vaginal Candidiasis	Vaginal epithelial cells	IL-1β, S100A8 alarmin	[79,80]
	Neutrophils	NET formation	[73]
Candidemia	Neutrophils	ROS and NET formation, JAGN1	[73,81]
	Platelets	IL-8, platelet microbicidal protein	[82,83]

## Data Availability

No new data were created or analyzed in this study. Data sharing is not applicable to this article.
